# Targeting lncRNA PVT1/miR‐145‐5p/ABCC‐1 Pathway Enhances Paclitaxel Sensitivity and Suppresses Melanoma Progression

**DOI:** 10.1155/ijog/2246468

**Published:** 2026-10-06

**Authors:** Negar Pedram, Reza Safaralizadeh, Mohammad Ali Hosseinpour Feizi, Dariush Shanehbandi

**Affiliations:** ^1^ Department of Animal Biology Faculty of Natural Sciences University of Tabriz Tabriz Iran tabrizu.ac.ir; ^2^ Immunology Research Center Tabriz University of Medical Sciences Tabriz Iran tbzmed.ac.ir

**Keywords:** apoptosis, combination therapy, lncRNA PVT1, melanoma, miR-145-5p, paclitaxel

## Abstract

**Background:**

As a highly aggressive type of skin cancer, melanoma cells show significant resistance against a variety of chemotherapeutics, such as paclitaxel, which limits the efficacy of the treatment. Studies have highlighted the potential role of noncoding RNA dysregulation in melanoma, affecting tumor progression and drug response, including lncRNA PVT1.

**Purpose:**

This study investigated whether simultaneous modulation of the lncRNA PVT1/miR‐145‐5p/ABCC1 axis enhances paclitaxel sensitivity and suppresses malignant behaviors in a melanoma cell line.

**Methods:**

Using SK‐MEL‐3 and A375 cells, we performed PVT1 knockdown using siPVT1, restored miR‐145‐5p function by transfection, and administered paclitaxel, either alone or in combination. Cell viability was assessed using the MTT assay; apoptosis and cell cycle distribution were analyzed by flow cytometry; and the expression of genes related to apoptosis, cell cycle regulation, migration, and drug resistance was quantified by qRT‐PCR.

**Results:**

Combination therapy markedly reduced cell viability compared with single treatments. Cotargeting PVT1 and miR‐145‐5p significantly enhanced paclitaxel‐induced apoptosis, evidenced by increased expression of proapoptotic and downregulation of antiapoptotic genes. The combination also induced a pronounced G1‐phase arrest with decreased levels of CDK‐4 and CDK‐6. Furthermore, gene expression analysis showed downregulation of MMP‐9 and ABCC1, along with upregulation of E‐cadherin, suggesting reduced migratory potential and reversal of drug resistance.

**Conclusions:**

Simultaneous PVT1 knockdown and miR‐145‐5p overexpression enhance the anticancer effects of paclitaxel by promoting apoptosis, inducing cell cycle arrest, and mitigating drug resistance in melanoma cells. Targeting the PVT1/miR‐145‐5p/ABCC1 regulatory network may provide a promising therapeutic strategy to overcome chemoresistance in melanoma.

## 1. Introduction

Melanoma is a highly aggressive form of skin cancer that originates from melanocytes. It poses a significant global health challenge due to its high metastatic potential and inherent resistance to conventional therapies [1]. Despite advancements in targeted treatments, drug resistance remains a major obstacle. This underscores the urgent need for novel therapeutic strategies that target critical survival pathways [2]. The incidence of melanoma has been steadily increasing for decades, particularly among fair‐skinned populations. Annually, increases ranging from 3% to 7%. Cutaneous melanoma has become the fastest growing cancer in the white population. Estimates suggest the incidence doubles every 10–20 years [3, 4]. These rates reflect the significance of the public‐health burden of melanoma.

Resistance to multiple anticancer agents is a major obstacle to the successful treatment of many types of malignant diseases [5]. Paclitaxel, a microtubule‐stabilizing chemotherapeutic agent, inhibits microtubule depolymerization and consequently induces G2/M phase arrest and apoptosis. Therefore, it is widely used in the treatment of several malignancies, including ovarian, breast, lung, and melanoma cancers. However, the clinical success of paclitaxel is often limited by the emergence of chemoresistance and dose‐dependent toxicity. Multidrug resistance is strongly associated with the overexpression of ATP‐binding cassette (ABC) transporters such as MRP1, which actively efflux chemotherapeutic compounds and reduce intracellular drug accumulation. Multidrug resistance‐associated protein 1 (MRP1), also known as ABCC1, is a member of the ABC transporter superfamily. These ABC membrane proteins utilize ATP hydrolysis to unidirectionally transport a wide range of molecules, including drugs and toxins, across the cell membrane [6]. MRP1, along with P‐glycoprotein (P‐gp) and breast cancer resistance protein (BCRP), is responsible for the efflux of many clinically important drugs, thereby leading to the multidrug resistance phenotype in cancer cells [7, 8]. Therefore, understanding the molecular regulators of ABCC1‐mediated efflux is essential for improving the chemosensitivity of melanoma cells.

Noncoding RNAs (ncRNAs), particularly long noncoding RNAs (lncRNAs) and microRNAs (miRNAs), contribute to these resistance mechanisms by regulating genes involved in apoptosis, cell cycle progression, epithelial–mesenchymal transition, and drug efflux. Their effect on drug resistance mechanisms differs from intrinsic cellular changes to extrinsic tumor microenvironment modulation. They play a central role in the maintenance of the stemness phenotype, which can induce cell survival and metastatic potential [9]. lncRNA PVT1, located on chromosome 8q24, is a well‐documented oncogenic lncRNA that is frequently overexpressed in various malignancies, including melanoma, esophageal cancer, and gastric cancer [10]. It exerts its oncogenic effects by influencing cellular processes such as proliferation, migration, invasion, and apoptosis, often by sponging tumor‐suppressor miRNAs [11]. Studies have shown that PVT1 downregulation inhibits cell proliferation and promotes apoptosis, whereas its overexpression increases proliferation in melanoma and colorectal cancer [12]. Furthermore, changes in Lnc‐PVT1 expression have been linked to chemotherapy resistance in cancer cells. For instance, in gastric cancer, lncRNA PVT1 can enhance cancer cell growth and suppress apoptosis by activating *Bcl2*, and its expression levels can predict resistance to 5‐fluorouracil [13]. High expression of lncRNA PVT1 is also a hallmark of cisplatin‐resistant colorectal cancer cells, correlating with increased proliferation, decreased apoptosis, and poor prognosis [13]. In clear cell renal cell carcinoma (ccRCC), lncRNA PVT1 upregulation is related to poor prognosis and cancer proliferation, and silencing PVT1 by siRNA transfection induces total apoptosis and cell cycle arrest at the G0/G1 phase by regulating the EGFR pathway. Their results were also confirmed by Western blotting findings [14].

Conversely, miR‐145‐5p is a recognized tumor‐suppressor miRNA that inhibits cancer cell growth, metastasis, and migration by directly targeting various oncogenes [15]. It was initially identified in mouse heart tissue and later in humans, showing a conserved sequence. Human miR‐145 (hsa‐miR‐145) is enriched in germline and mesodermal tissues and is located on chromosome 5 (5q32‐33) [16]. miR‐145‐5p functions by targeting various mRNAs and causing their downregulation. It acts as a vital regulator of genes that respond to chemotherapy in colorectal, gastric, and breast cancers [17–19]. Previous studies have shown that the expression level of miR‐145‐5p is decreased in breast cancer, bladder cancer, and melanoma cell lines, reinforcing its role as a tumor suppressor [20–22]. Importantly, miR‐145‐5p has been shown to increase the sensitivity of breast cancer cells to chemotherapy by downregulating the expression of proteins such as ABCC‐1 [5].

Although the individual roles of PVT1, miR‐145‐5p, and ABCC1 in cancer are increasingly understood, their combined interplay within the lncRNA PVT1/miR‐145‐5p/ABCC‐1 pathway, particularly in the context of paclitaxel chemotherapy for melanoma, warrants further investigation. To address this gap, the current study is aimed at investigating the changes in the expression of the lncRNA PVT1/miR‐145‐5p/ABCC‐1 gene pathway in melanoma. Specifically, we want to determine the regulatory dynamics of the PVT1/miR‐145‐5p/ABCC1 axis in melanoma cell lines and examine how modulation of this pathway influences cell viability, apoptosis, migration, and response to paclitaxel. By elucidating this regulatory network, we are aimed at identifying potential molecular targets for overcoming chemoresistance in melanoma.

## 2. Materials and Methods

### 2.1. Cell Line and Culture

The SK‐MEL‐3 and A375 human melanoma adenocarcinoma cell lines were obtained from the Pasteur Institute of Iran′s cell bank. Cells were cultured in RPMI‐1640 medium (Gibco, United States) supplemented with 10% fetal bovine serum (FBS) (Gibco, United States) and 1% penicillin/streptomycin (Gibco, United States). Cultures were maintained in a humidified incubator at 37°C with 5% CO_2_. Standard procedures for cell defrosting, freezing, storage, and passaging were followed, and cell counting was performed using a Neobar hemocytometer.

### 2.2. Transfection Protocol

For siPVT1 and miR‐145‐5p transfection, 500,000 cells per cuvette were prepared and mixed with 500 *μ*L of electroporation buffer. A total of 100 nM of siPVT1 or miR‐145‐5p was added, and electroporation was performed at 160 V for 25 ms using an electroporation device (Table S1). Cells were then seeded into appropriate plates for subsequent assays. The data obtained from real‐time quantitative PCR test results demonstrate transfection efficiency (Figure S1).

### 2.3. Cell Viability

Cell viability was assessed using the MTT colorimetric assay. Cells were seeded in 96‐well plates (10,000 cells/well). After 24 h, cells were treated with varying concentrations of paclitaxel (1–200 *μ*g/mL) to determine the IC_50_. For experimental groups, cells were treated with paclitaxel at its IC_25_ concentration, alone or in combination with siPVT1/miR‐145‐5p. The dose‐response curve for paclitaxel is demonstrated as Figure S2. After 24 h of treatment, 20 *μ*L of MTT solution (5 mg/mL) was added and incubated for 4 h. Formazan crystals were then dissolved with a sodium dodecyl sulfate/HCl buffer, and absorbance was read at 570 nm using an ELISA reader.

### 2.4. Apoptosis (Annexin V‐FITC/PI Staining)

Apoptosis was quantified using the annexin V‐FITC/PI apoptosis detection kit (Immunostep, Spain) according to the manufacturer′s instructions. Cells from all experimental groups were harvested after 24 h of treatment, washed with PBS, resuspended in binding buffer, and stained with annexin V‐FITC and propidium iodide. Samples were incubated in the dark for 15 min before analysis on a flow cytometer (Miltenyi, United States). The percentages of live, early apoptotic, late apoptotic, and necrotic cells were determined.

### 2.5. Cell Cycle Analysis (DAPI Staining)

Cell cycle distribution was analyzed using the DAPI staining kit (Sigma‐Aldrich, United States) and flow cytometry. Cells from experimental groups were harvested after 24 h of treatment, fixed with 75% ethanol at −20°C for 48 h, and then washed with cold PBS. Cells were treated with RNase A to remove RNA, followed by DAPI and Triton X‐100 staining. DNA content was analyzed using flow cytometry, and data were processed with FlowJo software.

### 2.6. Gene Expression (qRT‐PCR)

Total RNA was extracted from cell cultures using TRIzol reagent (Thermo Fisher Scientific, Waltham, Massachusetts, United States) according to the manufacturer′s instructions. RNA quantity and purity were assessed on a NanoDrop spectrophotometer by measuring A_600_/A_280_ and A_260_/A_230_ ratios. One microgram of total RNA was reverse‐transcribed into complementary DNA (cDNA) using the cDNA synthesis kit (Add Bio, South Korea) with random hexamer primers. qRT‐PCR reactions were carried out in a total volume of 20 *μ*L, containing 10 *μ*L of SYBR Green Master Mix, 0.5 *μ*L of each primer (forward and reverse), 1 *μ*L of template cDNA, and nuclease‐free water to volume. Reactions were run on a Step One Plus Real‐Time PCR System (Bio‐Rad, United States). GAPDH was used as the internal control for normalization, and relative expression levels were calculated using the 2^−*ΔΔ*CT^ method. Genes analyzed in this study included CDK‐6, CDK‐4, E‐cadherin, lncRNA PVT1, BAX, BCL‐2, MMP‐3, MMP‐9, MDR‐1 (ABCB1), caspase‐3, caspase‐8, caspase‐9, U6 snRNA, and miR‐145, and their primer sequences are provided in Table S2.

### 2.7. Statistical Analysis

All experiments were performed in triplicate, and results are presented as mean ± standard deviation (mean ± SD). Statistical analysis was performed using GraphPad Prism Version 6.1 and FlowJo Version 4.1 software. Prior to analysis, the assumption of normality was assessed using the Shapiro–Wilk test and homogeneity of variance was also evaluated using Levene′s test. Unpaired two‐tailed *t*‐tests were used for comparisons between exactly two groups (such as control vs. single treatment in qRT‐PCR and MTT assays). One‐way ANOVA was used for comparisons involving three or more independent groups with a single independent variable (treatment type), specifically for cell cycle phase percentages, apoptosis rates, and assessing gene expression levels. In addition, one‐way ANOVA followed by Tukey′s post hoc for all pairwise comparisons. A *p* value of < 0.05 was considered statistically significant, with significance levels reported as  ^∗^ *p* ≤ 0.05,  ^∗∗^ *p* ≤ 0.01,  ^∗∗∗^ *p* ≤ 0.001,  ^∗∗∗∗^ *p* ≤ 0.0001, and ns (nonsignificant) for *p* > 0.05.

## 3. Results

This study investigated the effects of lncRNA PVT1 siRNA, miR‐145‐5p, and paclitaxel, alone and in combination, on the viability, migration, apoptosis, and cell cycle of SK‐MEL‐3 and A375 melanoma cells.

### 3.1. Cell Viability Results

Although miR‐145‐5p and siPVT1 alone caused no significant change in viability, paclitaxel use and the combined treatments demonstrated a more pronounced cytotoxic effect. The miR‐145‐5p + siPVT1 + paclitaxel (combination) group showed the lowest viability in SK‐MEL‐3 cells, indicating a marked inhibitory effect compared with single treatments (*p* ≤ 0.0001 vs. control). The calculated IC_50_ values for paclitaxel were 45.3 ± 2.1 *μ* *g*/mL and 56 ± 5.6 *μ* *g*/mL in SK‐MEL‐3 and A375 cells, respectively. For the triple‐combination treatment, the IC_50_ values were 12.8 ± 1.2 *μ* *g*/mL and 40 ± 3.5 *μ* *g*/mL for SK‐MEL‐3 and A375 cells, respectively (Figure 1 and Figure 2).

**Figure 1:**
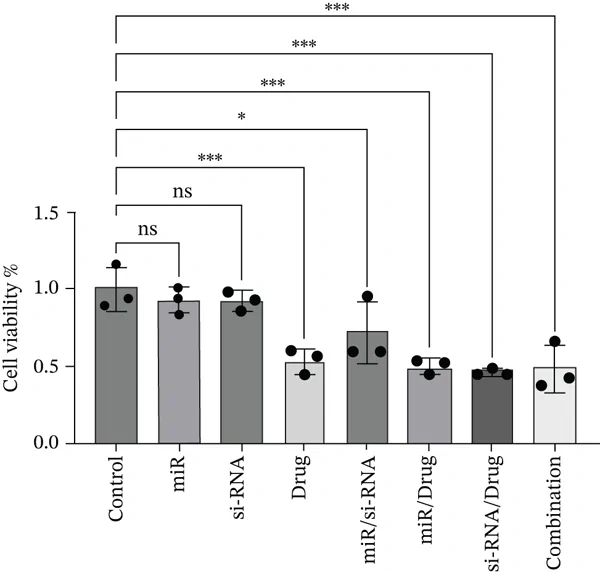
Cytotoxic test results in triplicate for miR‐145‐5p, siPVT1, paclitaxel, miRNA + siRNA combination, miRNA + paclitaxel combination, siRNA + paclitaxel combination, and triple‐combination treatment on SK‐MEL‐3 cell line (ns: *p* > 0.05,  ^∗^ *p* ≤ 0.05;  ^∗∗∗^ *p* ≤ 0.0001, *n* = 3).

**Figure 2:**
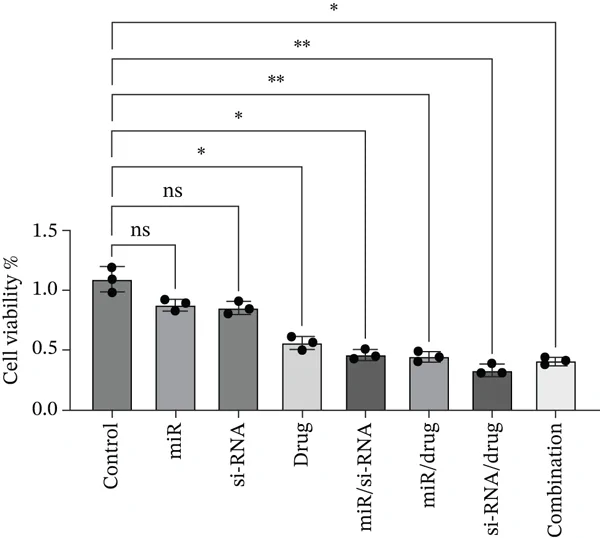
Cytotoxic test results in triplicate for miR‐145‐5p, siPVT1, paclitaxel, miRNA + siRNA combination, miRNA + paclitaxel combination, siRNA + paclitaxel combination, and triple‐combination treatment on A375 cell line (ns: *p* > 0.05,  ^∗^ *p* ≤ 0.05;  ^∗∗^ *p* ≤ 0.001, *n* = 3).

### 3.2. Apoptosis (Annexin V‐FITC/PI Staining)

Apoptosis in SK‐MEL‐3 and A375 melanoma cells was evaluated using annexin V‐FITC/PI staining followed by flow cytometry. Representative dot plots are shown in Figure 3 for SK‐MEL‐3 cells and Figure 4 for A375 melanoma cells, with quantitative data in Figure 5.

**Figure 3:**
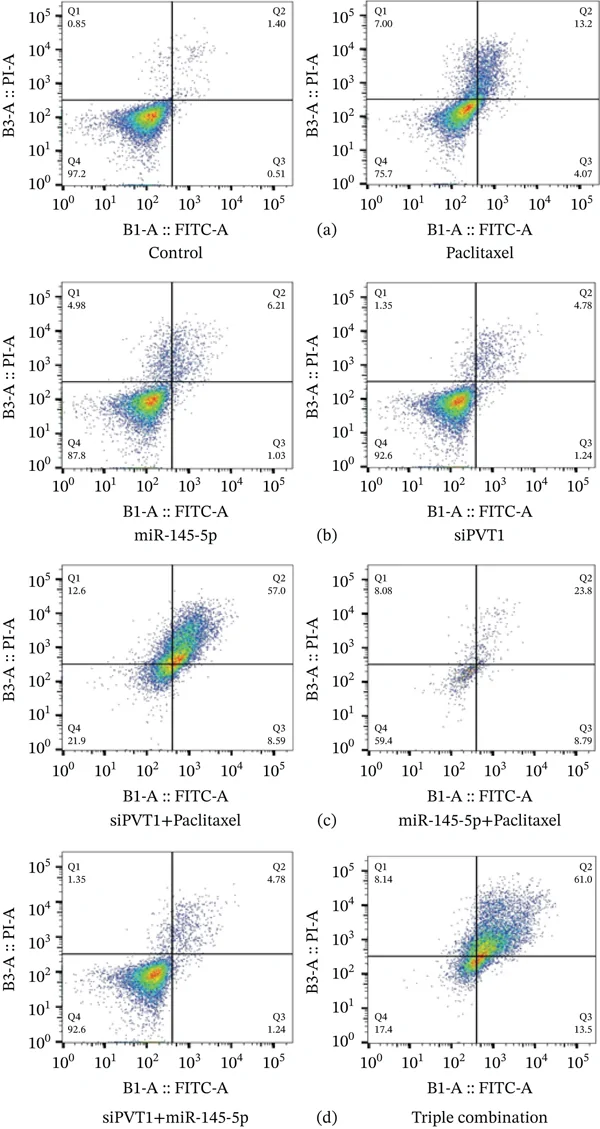
Representative flow cytometry dot plots illustrating apoptosis in SK‐MEL‐3 cells under various treatment conditions. The *x*‐axis represents annexin V‐FITC (B1‐A::FITC‐A) fluorescence intensity, and the *y*‐axis represents propidium iodide (B3‐A::PI‐A) fluorescence intensity. Numbers in each quadrant (Q1–Q4) indicate the percentage of cells in that specific population: Q4: live cells (annexin V−/PI−), Q3: early apoptotic cells (annexin V+/PI−), Q2: late apoptotic/necrotic cells (annexin V+/PI+), and Q1: necrotic cells (annexin V−/PI+). (a) Control and paclitaxel. (b) miR‐145‐5p and siPVT1. (c) siPVT1 + paclitaxel and miR − 145 − 5p + paclitaxel. (d) siPVT1 + miR − 145 − 5p and triple combination.

**Figure 4:**
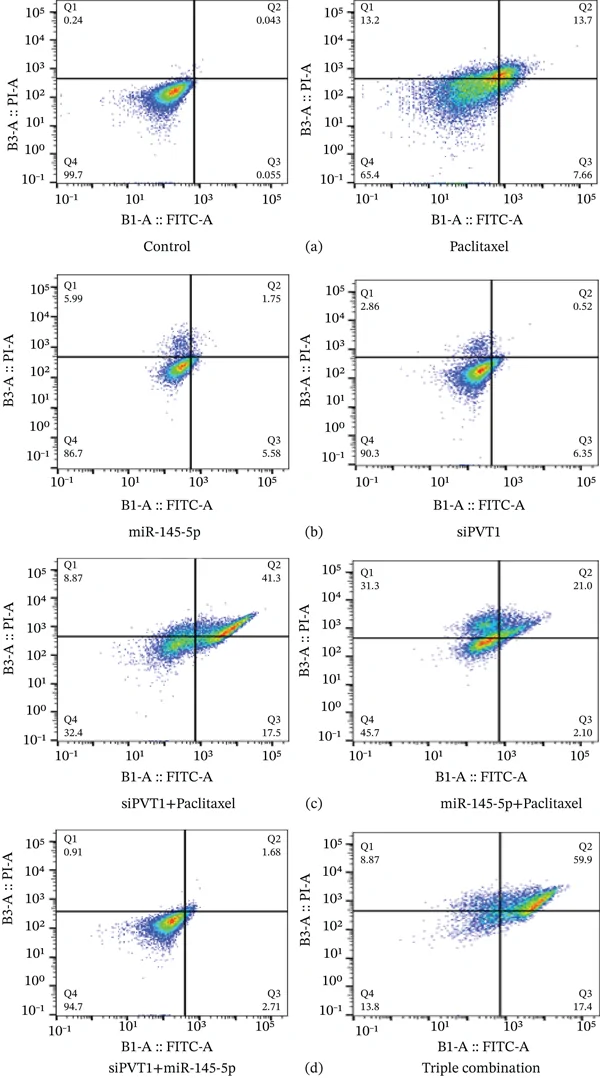
Representative flow cytometry dot plots illustrating apoptosis in A375 cells under various treatment conditions. The *x*‐axis represents annexin V‐FITC (B1‐A::FITC‐A) fluorescence intensity, and the *y*‐axis represents propidium iodide (B3‐A::PI‐A) fluorescence intensity. Numbers in each quadrant (Q1–Q4) indicate the percentage of cells in that specific population: Q4: live cells (annexin V−/PI−), Q3: early apoptotic cells (annexin V+/PI−), Q2: late apoptotic/necrotic cells (annexin V+/PI+), and Q1: necrotic cells (annexin V−/PI+). (a) Control and paclitaxel. (b) miR‐145‐5p and siPVT1. (c) siPVT1 + paclitaxel and miR − 145 − 5p + paclitaxel. (d) siPVT1 + miR − 145 − 5p and triple combination.

**Figure 5:**
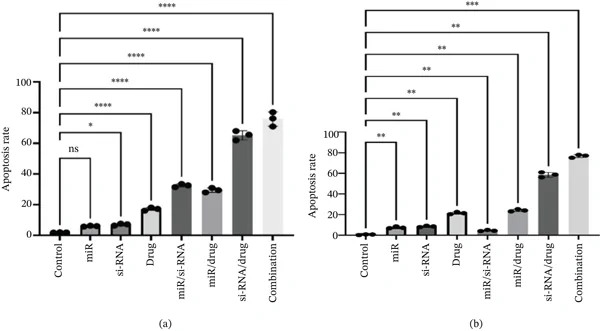
Quantification of apoptosis rate (percentage) across different treatment groups and control for (a) SK‐MEL‐3 and (b) A375 cell lines. Total apoptosis was calculated by summing the percentages of cells in the early apoptotic (Q3) and late apoptotic/necrotic (Q2) quadrants. Data are presented as mean ± standard error of the mean (SEM) (ns: *p* > 0.05,  ^∗^: *p* ≤ 0.05,  ^∗∗^: *p* ≤ 0.01,  ^∗∗∗^: *p* ≤ 0.001,  ^∗∗∗∗^: *p* ≤ 0.0001, *n* = 3).

The control group displayed a low basal apoptotic rate (1.91% apoptosis for SK‐MEL‐3 and about 0.1% for A375 cells) in both cell lines. Single treatments with siPVT1, miR‐145‐5p, or paclitaxel induced modest increases in apoptosis compared with the control, with paclitaxel alone resulting in approximately 17.27% total apoptosis for SK‐MEL‐3 and 21.36% for the A375 cell line. Dual combination treatments markedly enhanced apoptosis rates: siPVT1 + paclitaxel (siRNA and drug) achieved about 65.59% total apoptosis for SK‐MEL‐3 and 58.8% for A375 cell line, miR − 145 − 5p + paclitaxel (miR and drug) about 32.6% for SK‐MEL‐3 cells and 23.10% for A375 cells, and siPVT1 + miR − 145 − 5p (siRNA and miR) around 6% for SK‐MEL‐3 (*p* ≤ 0.0001 vs. single treatments), and 4.4% for A375 cells (*p* ≤ 0.01 vs. single treatments). Notably, the triple‐combination therapy (siPVT1 + miR − 145 − 5p + paclitaxel) induced the highest apoptotic rate of approximately 74.5% for SK‐MEL‐3 (*p* ≤ 0.0001) and 77.3% for the A375 cell line (*p* ≤ 0.001), representing a highly significant increase compared with all other groups. These results confirm a strong apoptotic response elicited by the combined modulation of PVT1 and miR‐145‐5p in conjunction with paclitaxel exposure.

### 3.3. Cell Cycle Analysis (DAPI Staining)

Cell cycle progression of SK‐MEL‐3 and A375 melanoma cells was analyzed using DAPI staining followed by flow cytometry after 24 h to determine the effects of single and combinatorial treatments. The representative cell cycle histograms for each group are shown in Figure 6 for the SK‐MEL‐3 cell line and in Figure 7 for the A375 cell line. The quantitative summary of G1 phase arrest for both cell lines is presented in Figure 8.

**Figure 6:**
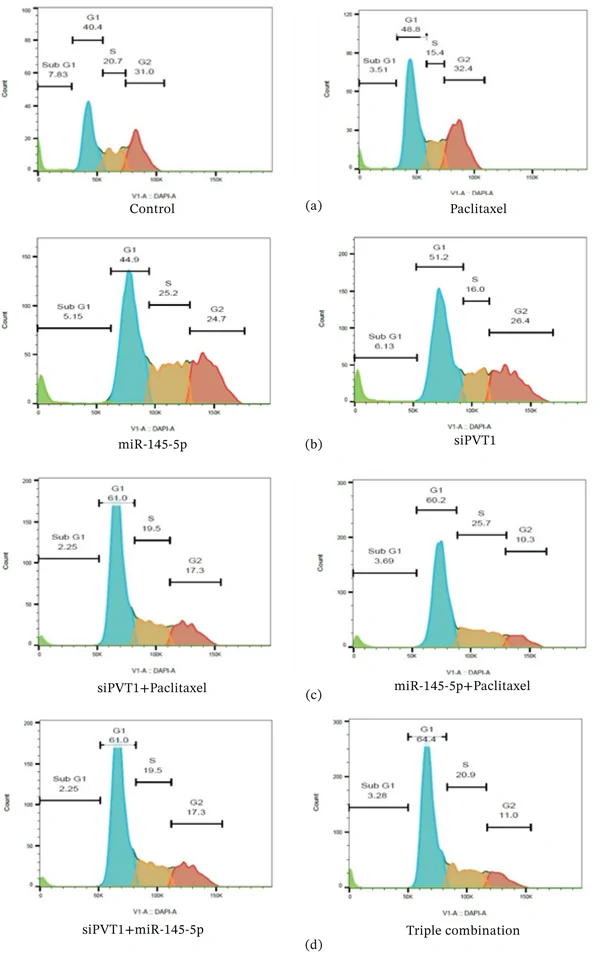
Representative flow cytometry histograms illustrating cell cycle distribution in SK‐MEL‐3 cells under various treatment conditions. The *x*‐axis (V1‐A::DAPI‐A) represents DAPI fluorescence intensity (DNA content), and the *y*‐axis (Count) represents cell count. Percentage values indicate the proportion of cells in sub‐G1 (apoptotic/subdiploid DNA content), G1, S, and G2/M phases. (a) Control and paclitaxel. (b) miR‐145‐5p and siPVT1. (c) siPVT1 + paclitaxel and miR − 145 − 5p + paclitaxel. (d) siPVT1 + miR − 145 − 5p and triple combination.

**Figure 7:**
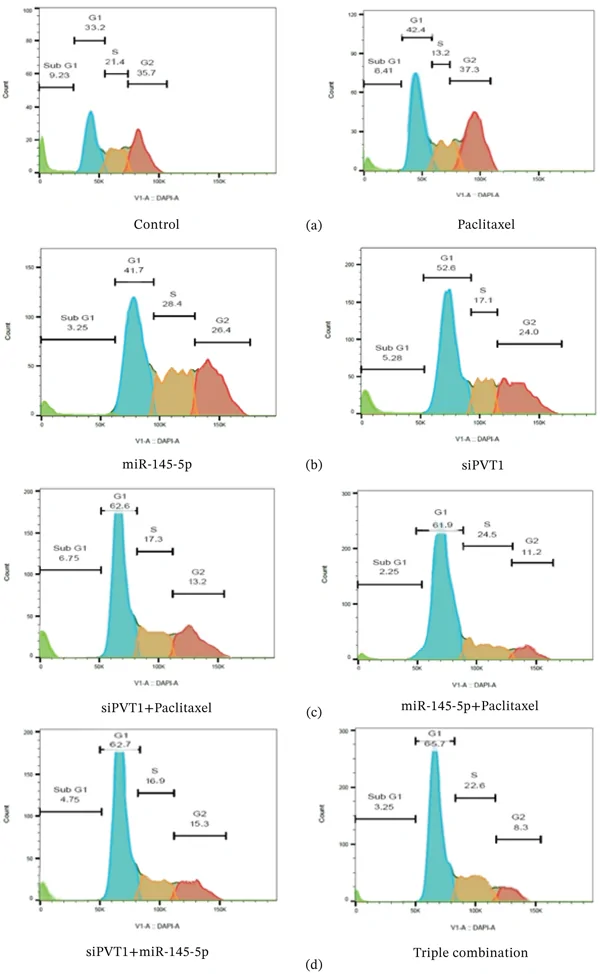
Representative flow cytometry histograms illustrating cell cycle distribution in A375 cells under various treatment conditions. The *x*‐axis (V1‐A::DAPI‐A) represents DAPI fluorescence intensity (DNA content), and the *y*‐axis (Count) represents cell count. Percentage values indicate the proportion of cells in sub‐G1 (apoptotic/subdiploid DNA content), G1, S, and G2/M phases. (a) Control and paclitaxel. (b) miR‐145‐5p and siPVT1. (c) siPVT1 + paclitaxel and miR − 145 − 5p + paclitaxel. (d) siPVT1 + miR − 145 − 5p and triple combination.

**Figure 8:**
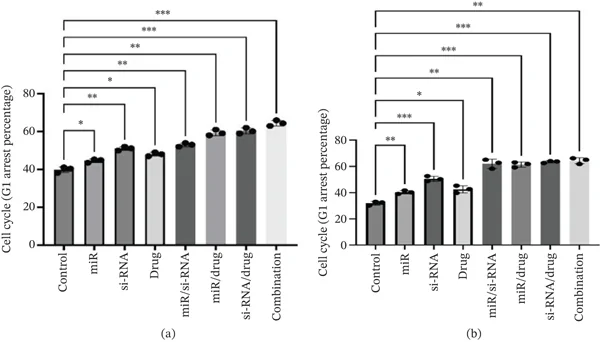
Quantification of G1 phase arrest percentage across different treatment groups and control for (a) SK‐MEL‐3 cells and (b) A375 cells. Data are presented as mean ± standard error of the mean (SEM) ( ^∗^: *p* ≤ 0.05,  ^∗∗^: *p* ≤ 0.01,  ^∗∗∗^: *p* ≤ 0.001, *n* = 3).

In SK‐MEL‐3 cells, control cultures showed 40.4% of cells in G1. Treatment with siPVT1 alone increased G1 to 51.2% (*p* ≤ 0.01) minor decrease in G2/M. miR‐145‐5p alone increased G1 to 44.9% (*p* ≤ 0.05) while reducing G2/M cell population. Paclitaxel monotherapy induced a modest but statistically significant increase in G1 (48.8%) and a concurrent increase in G2/M (18.7%) compared with control. Dual combinations further enhanced G1 arrest, with siRNA + drug, miR + drug, and siRNA + miR treatments showing around 61.0%, 60.2%, and 61.0% G1 phase cells, respectively, whereas G2/M fractions remained near or below control levels. The most pronounced effect was observed in the triple‐combination group (siRNA + miR + drug), which exhibited 64.4% of cells in the G1 phase, representing the highest level of arrest among all groups (*p* ≤ 0.0001 vs. control and single treatments) and the lowest G2/M (11.0%). Minor variations in the sub‐G1 fraction were also noted, ranging from 7.83% in the control to 3.28% in the combination group, consistent with apoptotic effects observed in previous assays. Overall, these findings demonstrate that the combinatorial treatments, particularly the triple combination, strongly enhance G1 phase arrest and inhibit the proliferation of SK‐MEL‐3 cells.

For A375 melanoma cells, following 24‐h treatment revealed similar results to SK‐MEL‐3 cells. The control group showed 33.2% of cells in the G1 phase. Single treatment with miR‐145‐5p increased G1 to 41.7% and reduced G2/M to 26.4%, whereas siPVT1 alone produced the strongest G1 arrest among single agents (52.6%) with only 24% of cells remaining in G2/M. Paclitaxel monotherapy increased G1 to 42.4% and G2/M to 37.3%, which represents a modest G2/M accumulation compared with control. Dual combinations further enhanced G1 arrest: miR − 145 − 5p + paclitaxel increased G1 to 61.9% with G2/M reduced to 11.2%, and siPVT1 + paclitaxel increased G1 to 62.6% with G2/M at 13.2%. The triple combination produced the highest G1 arrest (65.7%). These results demonstrate that in A375 cells, miR‐145‐5p and siPVT1 are potent inducers of G1 arrest.

### 3.4. Gene Expression Analysis

The mRNA expression levels of genes associated with apoptosis, cell cycle regulation, and drug resistance were analyzed for SK‐MEL‐3 cells, illustrated in Figure 9, and for A375 cells, illustrated in Figure 10, following different treatments.

**Figure 9:**
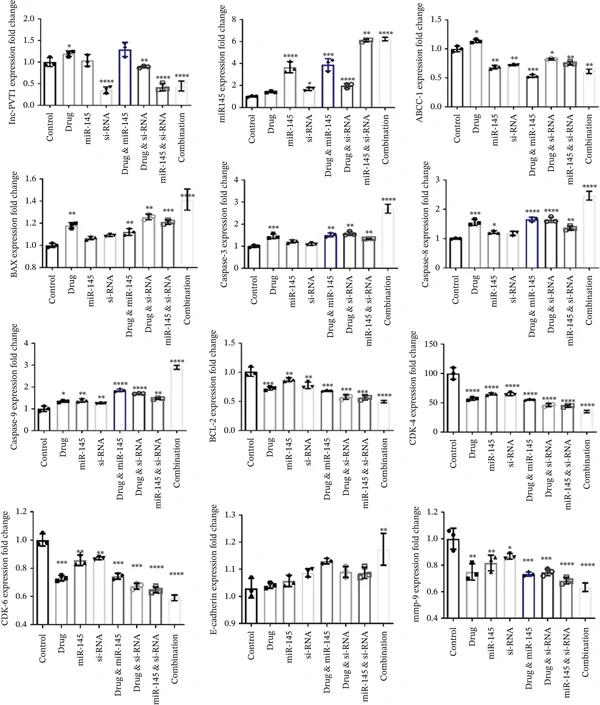
Expression analysis of genes involved in SK‐MEL‐3 cells following different treatments. Relative mRNA expression levels of apoptosis‐related (BAX, caspase‐3, caspase‐8, caspase‐9, BCL‐2), cell cycle–related (CDK‐4, CDK‐6), migration‐related (MMP‐9, E‐cadherin), drug resistance (ABCC1), and noncoding RNA (lncRNA PVT1, miR‐145‐5p) genes were assessed by quantitative real‐time PCR. Data are presented as mean ± SD of the three independent experiments. Statistical significance was determined relative to the control group ( ^∗^ *p* ≤ 0.05,  ^∗∗^ *p* ≤ 0.01,  ^∗∗∗^ *p* ≤ 0.001,  ^∗∗∗∗^ *p* ≤ 0.0001, *n* = 3).

**Figure 10:**
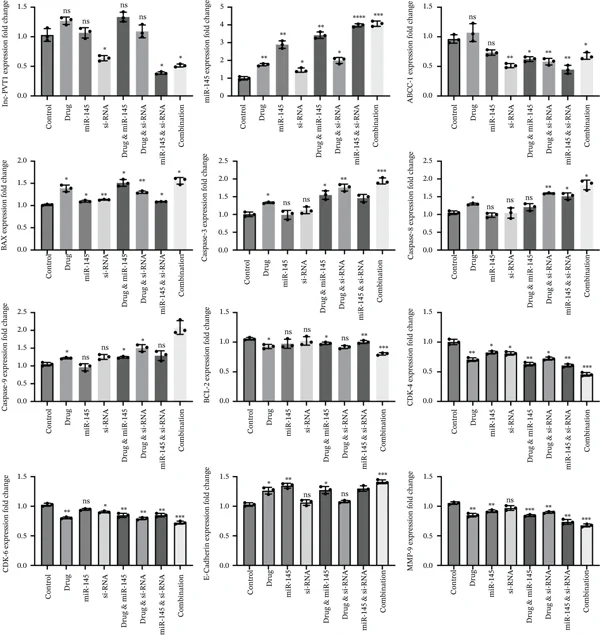
Expression analysis of genes involved in A375 cells following different treatments. Relative mRNA expression levels of apoptosis‐related (BAX, caspase‐3, caspase‐8, caspase‐9, BCL‐2), cell cycle–related (CDK‐4, CDK‐6), migration‐related (MMP‐9, E‐cadherin), drug resistance (ABCC1), and noncoding RNA (lncRNA PVT1, miR‐145‐5p) genes were assessed by quantitative real‐time PCR. Data are presented as mean ± SD of the three independent experiments. Statistical significance was determined relative to the control group ( ^∗^ *p* ≤ 0.05,  ^∗∗^ *p* ≤ 0.01,  ^∗∗∗^ *p* ≤ 0.001,  ^∗∗∗∗^ *p* ≤ 0.0001, *n* = 3).

Targeting the ncRNAs lncRNA PVT1 and miR‐145‐5p produced the intended regulatory effects. For the SK‐MEL‐3 cell line, lncRNA PVT1 expression was markedly downregulated in the siRNA and combination groups, whereas miR‐145‐5p expression was significantly upregulated in the miR‐145 and combination groups compared with the control. However, lncRNA PVT1 dysregulation was not significant in treated A375 cells. In addition, miR‐145‐5p expression was significantly upregulated in siRNA + miR‐treated cells.

The drug resistance gene ABCC1 showed a significant increase in expression in the paclitaxel group, indicating enhanced resistance under chemotherapy. However, the combination treatments, especially the triple combination, markedly reduced ABCC1 levels, suggesting that combined targeting of lncRNA PVT1 and miR‐145‐5p effectively mitigates drug resistance in the SK‐MEL‐3 cell line. For A375 cells, ABCC1 gene dysregulation did not show remarkable changes; however, siRNA and drug combination treatment suggest the efficacy of siPVT1 use.

Apoptosis‐related gene expression patterns supported the induction of programmed cell death by the combination treatment. The proapoptotic genes BAX, caspase‐3, caspase‐8, and caspase‐9 were significantly upregulated in the combination group in both cell lines, whereas the antiapoptotic gene BCL‐2 was downregulated compared with control and single treatments. These results align with flow cytometry findings indicating enhanced apoptotic activity.

Moreover, the combination therapy influenced genes related to cell cycle control and migration. The CDK‐4 and CDK‐6 genes were significantly downregulated in the combination group, consistent with the observed G1‐phase arrest. Additionally, MMP‐9 expression, a marker of extracellular matrix degradation and cell migration, was substantially decreased in the miRNA, dual, and combination groups, reaching its lowest level in the latter. Conversely, E‐cadherin expression was significantly elevated in the combination group, suggesting reinforced cell‐to‐cell adhesion and reduced migratory potential in both cell lines.

## 4. Discussion

Despite significant medical advancements, melanoma accounts for 80% of skin cancer–related mortality, largely due to its invasive characteristics and the development of drug resistance, which causes relapses after treatment [23]. Increasing evidence implicates that epigenetic abnormalities, such as DNA methylation, histone modification [24], ncRNAs expression profiles [25], and posttranslational modifications [26], play a central role in drug response, apoptosis, and metastasis in cancer regulation. The role of lncRNA PVT1, an important oncogenic lncRNA, in multidrug resistance is well documented. Zhang et al. [27] showed that siPVT1 transfection combined with cisplatin treatment in cisplatin‐resistant gastric cancer cells significantly reduced survival and increased apoptosis, concluding that PVT1 overexpression causes multidrug resistance and its knockdown reverses it. Yao et al. [28] further supported this by demonstrating that exosome‐derived circ‐PVT1 contributes to cisplatin resistance in gastric cancer cells. These findings collectively suggest that lncRNA PVT1 contributes to drug resistance by upregulating genes involved in multidrug resistance, and its suppression can re‐sensitize cancer cells to chemotherapy.

Although immune checkpoint inhibitors and BRAF/MEK–targeted therapies currently represent the standard of care for advanced melanoma [29], chemotherapeutic agents such as paclitaxel continue to be used in selected patients, particularly those with advanced or treatment‐refractory disease. Therefore, strategies aimed at overcoming paclitaxel resistance may still have clinical relevance [30]. Given the emerging evidence implicating PVT1 in cancer progression and therapeutic resistance [31], and miR‐145 crucial role in all aspects of cancer progression [32], further investigation is warranted to determine whether modulation of the PVT1/miR‐145‐5p axis may also enhance the efficacy of current targeted or immunotherapeutic approaches. Our findings reveal that simultaneous silencing of PVT1 by using siPVT1, overexpression of miR‐145‐5p, and paclitaxel administration enhances the cytotoxic and proapoptotic effects of paclitaxel in melanoma cells to a greater extent rather than any dual combination or single agent treatment.

The triple combined led to a substantial reduction in the survival and proliferation of melanoma cells, achieving the lowest cell viability among all treatment groups. Although dual combinations also reduced viability compared with single agents, the addition of the third component produced a marked benefit. Our findings are largely consistent with existing literature, as the observed suppression of lncRNA PVT1 by siRNA and its inhibitory effect on cancer cell proliferation and migration in melanoma cells aligns with previous research by Chen et al. [12]. Similarly, Ping et al. [33] showed that PVT1 knockdown increased cisplatin sensitivity in colorectal cancer cells, supporting our findings on enhanced drug sensitivity.

In terms of apoptosis, both siPVT1 and paclitaxel treatments individually increased apoptosis in cancer cells. However, the triple combination resulted in a more substantial increase in apoptosis. This was corroborated by our gene expression data, which revealed a significant upregulation of the proapoptotic genes (caspase‐3, caspase‐8, and caspase‐9), providing a molecular explanation for the observed increase in programmed cell death. The proapoptotic effect of PVT1 suppression observed in this study is corroborated by Bohosova et al. [34], who reported that PVT1 overexpression in kidney cell carcinoma affects apoptosis by upregulating MCL‐1, and its suppression leads to MCL‐1 reduction and apoptosis induction. Liu et al. [35] also demonstrated that PVT1 siRNA transfection combined with cisplatin treatment significantly reduced ovarian cancer cell survival and increased apoptosis, further solidifying the role of lncRNA PVT1 in regulating apoptosis.

Analysis of the cell cycle demonstrated that the triple combination produced the most pronounced G1 phase arrest with the lowest G2/M cell population, whereas siPVT1 and paclitaxel each induced modest G1 arrest. This was further supported by our gene expression results, which showed a significant downregulation of the cell cycle regulatory genes (CDK‐4 and CDK‐6) in the triple combination group. Paclitaxel mainly induces mitotic arrest of the cell cycle; however, its effect is highly concentration and cell‐type dependent [36]. In a study by Liao and Lieu [37], paclitaxel induced G1 and S phase arrest in U937 leukemic cells, rather than G2/M accumulation. Similarly, in human arterial smooth muscle cells, low‐dose paclitaxel caused primary G1 arrest [38], which is highly aligned with our finding. Even within melanoma models, A375 cells exhibit a high percentage of apoptotic cells in sub G0/G1 population with no significant change in G2/M cell population [39]. Critically, SK‐MEL‐3 cells have been reported to possess a weakened spindle checkpoint, rendering them less susceptible to paclitaxel‐induced mitotic arrest [40]. PVT1 silencing also prolongs the G0/G1 phase and reduces the S phase of the cell cycle, with its overexpression supporting cell cycle progression [12, 41]. These studies explain why the G1‐promoting effects of miR‐145‐5p and siPVT1 dominate the melanoma cells′ response rather than the predominant effect of paclitaxel alone.

The analysis of migration‐related gene expression revealed that the triple combination exerted a stronger inhibitory effect on genes associated with cell motility compared with single treatments. Transient upregulation of lncRNA PVT1 observed at 24‐h posttreatment (Figures 9 and 10) was considered a potential early stress response rather than a core resistance mechanism, especially given its lack of consistent significance across varied experimental conditions. Additionally, gene expression data showed a significant downregulation of the migration‐related gene MMP‐9 and an upregulation of the adhesion‐related gene E‐cadherin in the triple combination group. These molecular changes suggest that the triple treatment may reduce the migratory and invasive potential of melanoma cells. The observed changes in migration‐ and invasion‐associated molecular markers following PVT1 knockdown in this study are also consistent with findings by Gong et al. [42] in glioma cells. In ovarian cancer, Dong et al. [43] showed that PVT1 knockdown markedly reduced proliferation, migration, and invasion in SKOV3 and CAOV3 cells. In glioma, Han et al. [44] demonstrated that PVT1 knockdown inhibited cell viability, migration, and invasiveness in U251 and U87 cells.

Moreover, the expression of the MDR1 gene, a key mediator of drug resistance also known as ABCC1, was significantly decreased following triple combination treatment in both cell lines; the effect was more pronounced in SK‐MEL‐3 cells (*p* < 0.01) than in A375 cells (*p* < 0.05). However, ABCC1 expression was not remarkable in A375 treated with paclitaxel and miR‐145‐5p. This quantitative difference may reflect inherent molecular differences between the two cell lines and variations in the regulatory circuitry linking PVT1/miR‐145‐5p to ABCC1. This ABCC1 expression profile, however, suggests that the triple combination therapy effectively mitigates drug resistance mechanisms in melanoma cells.

Furthermore, miR‐145‐5p transfection alone reduced lncRNA PVT1 expression and increased apoptosis. When combined with paclitaxel, miR‐145‐5p showed a significant reduction in lncRNA PVT1 expression and further increased apoptosis. This supports miR‐145′s role as a tumor suppressor and its ability to enhance the effects of chemotherapy. The study also found that miR‐145‐5p affected the expression of genes such as CDK‐6, CDK‐4, E‐cadherin, MMP‐9, MDR‐1, Bcl‐2, Bax, caspase‐3, caspase‐8, and caspase‐9, further elucidating its broad regulatory impact on cancer hallmarks.

The interaction between miR‐145‐5p and lncRNA PVT1 is also supported by previous studies. Qu et al. [45] reported that lncRNA PVT1 is upregulated in breast cancer and promotes glycolysis, proliferation, migration, and invasion by competitively binding (sponging) endogenous miR‐145‐5p; conversely, PVT1 knockdown or restoration/overexpression of miR‐145‐5p reversed these effects. They further reported a negative correlation between PVT1 and miR‐145‐5p in breast cancer cells. The mechanism by which miR‐145‐5p enhances chemotherapy sensitivity, particularly through targeting ABCC1, is supported by Zheng and Xu [46], who demonstrated ABCC1 as a direct target of miR‐145‐5p. As the miR‐145‐5p enhancement restores the effect of circPVT1 knockdown on chemotherapy resistance. At the molecular level, lncRNA PVT1 acts as a competitive endogenous RNA (ceRNA) by containing binding motifs complementary to the seed region of miR‐145‐5p. This sponging effect reduces the availability of miR‐145‐5p to interact with its downstream targets, such as ABCC1, which is responsible for drug efflux. These results suggest that PVT1 indirectly upregulates ABCC1 expression by sequestering miR‐145‐5p, leading to increased drug efflux and decreased intracellular drug accumulation, thereby promoting chemoresistance [47, 48].

The observed increase in apoptosis due to miR‐145‐5p transfection aligns with Wei et al. [49], who showed that suppressing PVT1 through the miR‐145‐5p/ITGB8 axis induced apoptosis in nonsmall cell lung cancer cells, and that miR‐145‐5p significantly inhibited proliferation, migration, and invasion in cervical cancer cells by targeting lncRNA UCA1. Pan et al. [50] found that overexpression of miR‐145 suppressed proliferation, migration, and invasion and promoted apoptosis. Specifically, they observed that miR‐145 upregulation decreased Bcl‐2, increased Bax, thereby elevating the Bax/Bcl‐2 ratio, and activated the caspase‐3 cascade and PARP cleavage.

Overall, our findings indicated that the triple combination treatment (PVT1 siRNA, miR‐145, and paclitaxel) yielded the most favorable outcomes in terms of increased apoptosis and modulation of molecular markers associated with reduced cell migration and invasion potential compared with other treatments and the control group. This suggests a potent combinatorial effect to overcome drug resistance in melanoma when a combination of gene therapy (siRNA and miR‐145‐5p) and chemotherapy (paclitaxel) is applied. This combinatorial effect indicates that targeting multiple pathways simultaneously can enhance therapeutic efficacy and potentially circumvent resistance mechanisms that often limit the success of single‐agent therapies.

## 5. Limitations and Future Directions

Building upon these promising findings and considering the study′s limitations, several avenues for future research are proposed to further validate and expand upon the current study′s conclusions to establish therapeutic potential and safety. Investigate the combined effect of PVT1 siRNA, miR‐145‐5p, and paclitaxel in other melanoma cell lines, as well as in other types of cancer cell lines, to assess the generalizability of these findings. Evaluating the combined effect of PVT1 siRNA, miR‐145‐5p, and paclitaxel with other common chemotherapeutic drugs or alternative treatment methods, such as radiotherapy, to explore broader therapeutic strategies. These experiments were conducted using in vitro cell line models. Although the used cell lines are representative of melanoma, they do not fully recapitulate the complex in vivo tumor microenvironment, which includes interactions with the immune system, extracellular matrix, and vascularization. Therefore, the potent combinatorial antitumor effects observed in vitro may not directly translate to the same magnitude or efficacy in a clinical setting. Future research should focus on validating these findings through in vivo studies using appropriate animal models. Such studies are crucial for assessing the drug′s efficacy, pharmacokinetics, and potential systemic side effects in a more physiologically relevant context.

In addition, future studies should include protein‐level and mechanistic validation, such as Western blot and dual‐luciferase reporter assays, to further confirm the regulatory interactions within the PVT1/miR‐145‐5p/ABCC1 axis. Quantitative drug combination analyses should also be performed to determine whether the observed combinational effects represent true pharmacological synergy. In addition, the effects of the combined treatment on melanoma cell migration and invasion should be validated using functional assays, such as wound‐healing, Transwell migration, and Matrigel invasion assays.

## 6. Conclusion

In vitro results suggest that combined targeting of the lncRNA PVT1/miR‐145‐5p/ABCC1 axis together with paclitaxel may produce a potent, combinatorial antitumor effect on melanoma cells. Specifically, simultaneous PVT1 knockdown and miR‐145‐5p restoration enhanced paclitaxel sensitivity, markedly increased apoptotic cell death, induced G1 cell‐cycle arrest, and downregulated migration‐related and drug‐resistance genes (including ABCC1/MRP1). These findings suggest that cotargeting ncRNA networks that control drug efflux and survival signaling can sensitize melanoma cells to chemotherapy and may represent a promising strategy to overcome chemoresistance. Although our in vitro results are promising, they represent a preliminary step. Further validation and optimization are necessary before considering clinical translation.

## Author Contributions

Negar Pedram: methodology, formal analysis, investigation, and writing—original draft preparation. Reza Safaralizadeh: conceptualization, formal analysis, investigation, writing—review and editing, and supervision. Dariush Shanehbandi: conceptualization, methodology, formal analysis, investigation, writing—original draft preparation, writing—review and editing, and supervision. Mohammad Ali Hosseinpour Feizi: writing, review and editing, and supervision.

## Funding

No funding was received for this manuscript.

## Disclosure

All authors read and approved the final manuscript.

## Conflicts of Interest

The authors declare no conflicts of interest.

## Supporting information


**Supporting Information** Additional supporting information can be found online in the Supporting Information section. Figure S1: The real‐time quantitative PCR results showing transfection efficiency for siPVT1 and microRNA‐145‐5p. Figure S2: Dose‐response curve data for SK‐MEL‐3 cells exposed to different paclitaxel concentrations. Table S1: siRNA sequences and electroporation parameters. Table S2: Primer sequences used for qRT‐PCR.

## Data Availability

The data that support the findings of this study are available from the corresponding author upon reasonable request.
