# Supplementary material for: Targeting lncRNA PVT1/miR‐145‐5p/ABCC‐1 Pathway Enhances Paclitaxel Sensitivity and Suppresses Melanoma Progression
Source: Int J Genomics. 2026 Oct 6;2026:2246468. doi: 10.1155/ijog/2246468 (PMC13639832; doi:10.1155/ijog/2246468)
Supplement: Supplementary file 1 [file IJOG-2026-2246468-s001.docx]

**Supplementary Figure 1** The real-time quantitative PCR results showing transfection efficiency for siPVT1 and microRNA-145-5p.

**Supplementary Figure 2** dose-response curve data for SK-MEL-3 cells exposed to different paclitaxel concentrations**.**

**Supplementary Table 1** siRNA Sequences and Electroporation Parameters.

| Parameter | Value |
| --- | --- |
| siRNA Sense Sequence | GCTGTCAAAGAGGCCTGAAGA |
| siRNA Antisense Sequence | TCTTCAGGCCTCTTTGACAGC |
| Electroporator | BTX ECM 630 and Bio-Rad Gene Pulser |
| Voltage | 160 V |
| Resistance | 100 Ω |
| Capacitance | 25 μF |
| Typical Time Constant | ~25 milliseconds |

**Supplementary Table 2** Primer sequences used for qRT‑PCR.

| Target | Forward primer (5′→3′) | Reverse primer (5′→3′) | TM (°C) |
| --- | --- | --- | --- |
| CDK‑6 | AGAAGAAGACTGGCCCTAGAG | TGGAAGTATGGGTGAGACAGG | 60 |
| CDK‑4 | AGCTTGCGCCTGTCTCTATG | GATCAAGGGAGACCCTCAGCC | 60 |
| E‑Cadherin | GGTTATTCTCCC CATCAGCT | CTTTGGCTGAGGATGGTGTA | 60 |
| lncRNA PVT1 | TGA GAA CTG TCC TTA CGT GAC | AGA GCACCA AGA CTG GCT CT | 60 |
| BAX | TTTGCTTCAGGGTTTCATCCA | TCTGCAGTCCATGTTACTGTG | 60 |
| BCL‑2 | GAGTTCGGTGGGGTCATGTG | CACCTACCCAGCACCCAGTTA | 62 |
| MMP‑3 | TACTGGAGATTTGATGAGAAGAG | TACAGATTCACGCTCAAGTTCC | 60 |
| MMP‑9 | ATTCATCTTCCAAGGCCAATCC | CTTGTCGCTGTCAAAGTTCG | 60 |
| MDR-1 | TTCCGCTTCTTCGTCTGCTT | TCTTGCCATCTTCCGACCAC | 60 |
| GAPDH | AACATCATCCCTGCCTCTAC | CTGCTTCACCACCTTCTTG | 60 |
| Caspase3 | ATGGTTTGAGCCTGAGCAGA | GGCAGCATCATCCACACATAC- | 60 |
| Caspase9 | GCAGGCTCTGGATCTCGGC | GCTGCTTGCCTGTTAGTTCG | 60 |
| Caspase8 | ACCTTGTGTCTGAGCTGGTCT | GCCCACTGGTATTCCTCAGGC | 60 |
| U6 | GCTTCGGCAGCACATATAТАСТААAT | CGCTTCACGAATTTGCGTGTCAT | 60 |
| miR-145 | TTTGGTAGGAGATTGGGGAATAT | СТСТТСТАCATCCAACCCCATСТА | 60 |
